# *Drosophila* Exhibit Divergent Sex-Based Responses in Transcription and Motor Function After Traumatic Brain Injury

**DOI:** 10.3389/fneur.2020.00511

**Published:** 2020-06-19

**Authors:** Ekta J. Shah, Katherine Gurdziel, Douglas M. Ruden

**Affiliations:** ^1^Department of Pharmacology, Wayne State University School of Medicine, Detroit, MI, United States; ^2^Office of the Vice President for Research, Wayne State University, Detroit, MI, United States; ^3^Institute of Environmental Health Sciences, Wayne State University, Detroit, MI, United States

**Keywords:** TBI, immune, mitochondria, sex-differences, bimodal recovery

## Abstract

Every year, millions of people in the US suffer brain damage from mild to severe traumatic brain injuries (TBI) that result from a sudden impact to the head. Despite TBI being a leading cause of death and disability worldwide, sex differences that contribute to varied outcomes post-injury are not extensively studied and therefore, poorly understood. In this study, we aimed to explore biological sex as a variable influencing response to TBI using *Drosophila melanogaster* as a model, since flies have been shown to exhibit symptoms commonly seen in other mammalian models of TBI. After inflicting TBI using the high-impact trauma device, we isolated *w*^1118^ fly brains and assessed gene transcription changes in male and female flies at control and 1, 2, and 4 hr after TBI. Our results suggest that overall, *Drosophila* females show more gene transcript changes than males. Females also exhibit upregulated expression changes in immune response and mitochondrial genes across all time-points. In addition, we looked at the impact of injury on mitochondrial health and motor function in both sexes before and after injury. Although both sexes report similar changes in mitochondrial oxidation and negative geotaxis, locomotor activity appears to be more impaired in males than females. These data suggest that sex-differences not only influence the response to TBI but also contribute to varied outcomes post-injury.

## Introduction

Traumatic brain injuries (TBI), sudden jolts to the head that cause brain damage (1), can result from sports, domestic violence, auto accidents, falls or explosive blasts (2, 3). TBI is an emerging health epidemic with ~2.5 million cases occurring annually that are severe enough to require hospitalization or cause death (4). Although there is growing evidence that TBI outcome is influenced by host genotype and sex (5), research has largely overlooked investigating sex of the patient as a contributing factor to varied outcomes between males and females. For instance, fewer women than men are recruited for clinical trials and male animal models are predominantly used in TBI research (6). In addition to the differences in how men and women may acquire injuries, there are also sex-specific hormones that affect outcome to TBI (7). Brain function, pharmacokinetics and cellular pathways are all influenced by biological sex (6), therefore, consideration of sex as a variable is crucial for development of successful treatments.

Research that does exist regarding sex differences in TBI outcomes suggests females may be more affected than males (8). Neurocognitive computerized testing in college athletes who sustained sports-related concussions, showed females were 1.7 times more frequently cognitively impaired than males following injury (9). A meta-analysis of 8 TBI studies (20 outcome variables) reported that women were worse than men for 85% of the measured variables (8). Although no differences were seen in neurodegeneration, blood-brain barrier alteration or microglia activation between male and female adult mice after a moderate controlled cortical impact showed that females exhibited more astrocytic hypertrophy at 1-day post-injury (10). Male and female rats given either a single mild TBI or repetitive mild TBI (rmTBI) using a lateral impact model exhibit a sex-dependent response to trauma. All rats that were given rmTBI showed balance, locomotion and motor coordination deficits, but only males had short-term working memory deficits and only females had increased depressive-like behavior in response to rmTBI (11). In an attempt to study the effects of hormone levels, controlled cortical impact was performed on adult rats and endogenous hormones measured by gas chromatography/mass spectrometry. Increased levels of corticosterone, indicative of acute stress was seen in both sexes whereas an increase in progesterone and its metabolites varied by sex, time and location of injury (12). In spite of growing evidence that points toward sex-dependent changes in response to TBI, inclusion of both sexes in all preclinical TBI research is not widely conducted.

Brain injury after TBI typically occurs in 2 stages resulting in primary and secondary damage (13). Primary damage which starts at the moment of impact, resulting from the brain crashing back and forth inside the skull causing bruising, bleeding or even skull fractures, can involve the entire brain or be isolated to a specific part (14, 15). Secondary damage which can continue over several weeks, months or even years (14) after the TBI event is characterized by disruption of cellular processes (16), membrane depolarization, excessive release of excitatory neurotransmitters, activation of NMDA and Ca^2+^ and Na^+^ channels (13). Secondary damage also results in activation of apoptotic and inflammatory pathways, mitochondrial dysfunction, over-production of reactive oxygen species and structural changes in biological membranes (13, 17). The most pronounced effect of TBI is axonal damage which when coupled with brain injury triggers a cascade of events increasing tau phosphorylation and neurofibrillary tangle formation (18–20). Intriguingly, the expression of hyperphosphorylated human *tau* (hTau) has also been shown to elongate mitochondria resulting in mitochondrial dysfunction and cell-death, suggesting a possible cause of mitochondrial abnormalities that have been implicated in neurodegenerative disorders (21). Accumulation of several neurodegeneration-related proteins like synuclein, amyloid-beta, tau, TAR DNA-binding protein 43, presenilin and ubiquitin is also seen post-TBI (22–24).

In this study, we are using *Drosophila melanogaster* as a model to study TBI. The complex brain and nervous system of flies make it a very powerful model for neuroscience research (25, 26). Consistent with mammalian and human TBI studies, flies subjected to rapid acceleration and impact exhibit TBI related secondary phase symptoms including innate immune response, neurodegeneration, disrupted sleep cycles and a decreased lifespan (27, 28). The few studies that looked at responses to TBI in *Drosophila* have reported data either from one sex only (29, 30), both sexes combined (31) or only studied epigenetic changes in offspring after injury in both sexes (32). Therefore, we sought to compare response to traumatic injury in both male and female fly brains.

*w*^1118^ male and female flies were inflicted with full body trauma using the high-impact trauma (HIT) device and brains were isolated for further analysis. We report changes in gene expression and motor function in both sexes 1, 2, and 4 hr after TBI and show that transcriptional changes in *Drosophila* females are more pronounced than males. In addition, both sexes show effects on motor function in response to TBI. To the best of our knowledge, this is the first study to report changes in gene transcription at immediate time-points post-injury and to do so in both sexes.

## Materials and Methods

### Fly Stocks and Crosses

All fly stocks were stored at 25°C at constant humidity and fed with standard sugar/yeast/agar medium. *w*^1118^ and *UAS-MitoTimer* (#57323) were obtained from the Bloomington *Drosophila* Stock Center. *elav-Gal4* (#458) was obtained from Dr. Sokol Todi (Wayne State University). *UAS-MitoTimer* and *elav-Gal4* crosses were performed at the conditions described above. All assays were performed on adult mated flies (10–14 days old).

### Traumatic Brain Injury (TBI)

Full body trauma from a single strike of a modified high impact trauma (HIT) device with the impact arm constrained to a 45° angle was used to inflict male and female files with TBI (27, 33). No more than 50 flies were placed in a plastic vial before being confined to the bottom quarter of the vial by a stationary cotton ball. When the spring was deflected and released, the vial rapidly contacted the pad delivering trauma to the flies as they contact the vial wall.

### MitoTimer

Mitochondrial oxidation was assessed using a modified MitoTimer protocol (34). Brains were dissected from either control or TBI flies expressing MitoTimer. Three replicates of 10 brains per condition were placed in each well of a 96-well clear-bottom plate containing 50 μl 1XPBS. Red and green fluorescence were measured immediately after dissecting brains for each time-point at the excitation/emission wavelengths of 543/572 and 485/528, respectively, using the Synergy H1 microplate reader (Biotek). The ratio of red over green fluorescence was reported as an indicator of the level of mitochondrial oxidation. All data are represented as mean ± standard error of the mean (SEM). All statistical analyses were performed using GraphPad Prism. One-way ANOVA with Dunnett's multiple comparisons test was used to compute statistical significance (*p* < 0.05) between groups.

### RNA Isolation

Total RNA was extracted from single fly brains using the QIAzol® lysis reagent and Direct-zol™ RNA MicroPrep kit (Zymo Research) following manufacturer's instructions.

### 3′ mRNA Expression Analysis

Expression analysis was conducted in collaboration with the Wayne State University Genome Sciences Core. Three biological replicates were used for each condition.

QuantSeq 3′ mRNA-Seq Library Prep Kit FWD for Illumina (Lexogen) was used to generate libraries of sequences close to 3′ end of polyadenylated RNA from 15 ng of total RNA in 5 μl of nuclease-free water following low-input protocol. Library aliquots were assessed for overall quality using the ScreenTape for the Agilent 2200 TapeStation and quantified using Qubit™ 1X dsDNA HS Assay kit (Invitrogen). Barcoded libraries were normalized to 2 nM before sequencing at 300 pM on one lane of a NovaSeq 6000 SP flow cell. After de-multiplexing with Illumina's CASAVA 1.8.2 software, the 50 bp reads were aligned to the *Drosophila* genome (Build dm3) with STAR_2.4 (35) and tabulated for each gene region (36). Differential gene expression analysis was used to compare transcriptome changes between conditions using edgeR v.3.22.3 (37) and transcripts were defined as significantly differentially expressed at absolute log_2_ fold change (|log_2_FC|) > 1 with an FDR <0.05. Significant gene expression changes were submitted for gene ontology analyses using RDAVID (38) for the following categories: GOTERM_BP_ALL, GOTERM_MF_ALL, UP_KEYWORDS, GOTERM_BP_DIRECT, and GOTERM_MF_DIRECT.

### Heatmaps

Heatmaps were generated using Java Treeview (39). Counts representing the number of reads mapped to each gene were obtained using HTSeq (36) on STAR alignments (35) before normalization. To normalize, a scaling factor was determined by dividing the uniquely mapped reads for each sample by the sample with the highest uniquely mapped number of reads. Each gene count for all samples was multiplied with this scaling factor for normalization. Log_2_ of the normalized averaged counts for all 3 replicates is represented for each condition on the orange scale (0–10). The log_2_ fold change, represented on yellow-blue scale (0–6), for each gene is obtained from differential expression analysis across all 3 replicates (37). Genes significant (|log_2_ FC| > 1, *p* < 0.05) in at least 1 time point are indicated in black text.

### Quantitative Real Time PCR

qRT-PCR was performed on select genes to validate 3′ mRNA-Seq results. Total RNA was isolated from 10 fly brains for males and females at control and 2 hr after TBI as described in RNA isolation. To measure the expression levels of target genes, 2 ng RNA was mixed with TaqMan™ Gene Expression Primers (Thermo Fisher, Waltham, MA, USA) and TaqMan™ RNA-to-C${}_{\text{T}}^{™}$ 1-Step Kit (Thermo Fisher, Waltham, MA, USA). qRT-PCR reactions were run in a 384-well plate containing 2 biological and 3 technical replicates of each condition. *Drs* (Dm01822006_s1), *DptB* (Dm01821557_g1), *Mtk* (Dm01821460_s1), *mRpL55* (Dm02142138_g1), and *AttA* (Dm02362218_s1) were quantified with QuantStudio 12K Flex run to 40 cycles, using 2^−ΔΔ*Ct*^ (cycle threshold) methods and normalizing all transcripts to the reference gene, *RpL32* (Dm02151827_g1). Significant change (*p* < 0.05) was computed using 2-tailed Student's *t*-test for unequal variance.

### Climbing Assay

Negative geotaxis was measured using a modified climbing assay protocol (31, 40–42). Approximately 20 flies per condition were placed in plastic vials. Flies were gently tapped to the bottom of the vials and then the number of flies that climbed above a 7 cm mark within 15 sec were recorded. The assay was carried out in triplicate (60 flies total) for each of the following conditions: 10 min after flies were inflicted with trauma for immediate response with measurements repeated at 24, 48, and 72 hr. The average percent climbed across all 3 replicates is reported as mean ± SEM. Flies were maintained at 25°C for the duration of the assay. Mixed design ANOVA was used to compute significance (*p* < 0.05) with condition (Control or TBI) as a between-subjects factor, time (10 min, 24, 48, and 72 hr) as a within-subjects factor and vial as random factor with Tukey for *post-hoc* comparison. The mixed design ANOVA was performed in R Core Team (43) using the “emmeans” package.

### Locomotor Activity Assay

To measure locomotor activity, individual flies (24 biological replicates/condition) for control and TBI condition were placed in tubes containing regular fly food in a Drosophila activity monitoring system which measures the number of times a given fly crosses an infrared beam (TriKinetics Inc., Waltham, MA) (44). The activity was assessed for 2 days. Flies were subjected to 12-hr light/dark cycles with activity summarized every 30 min producing 96 timepoints of data. The number of beam breaks occurring as a result of fly movement in 30-min time-bins before the specified time-point are plotted as locomotor activity for that time-point. Flies that did not live through the recording period were not used in the calculations. Repeated measures ANOVA with Fisher's Least Significant Difference (LSD) and Bonferroni for multiple comparisons test was used to compute statistical significance (*p* < 0.05) between control and TBI groups using SPSS.

## Results

### Identification of Sex Dependent Gene Expression Changes in Response to TBI

**Figure 1:**
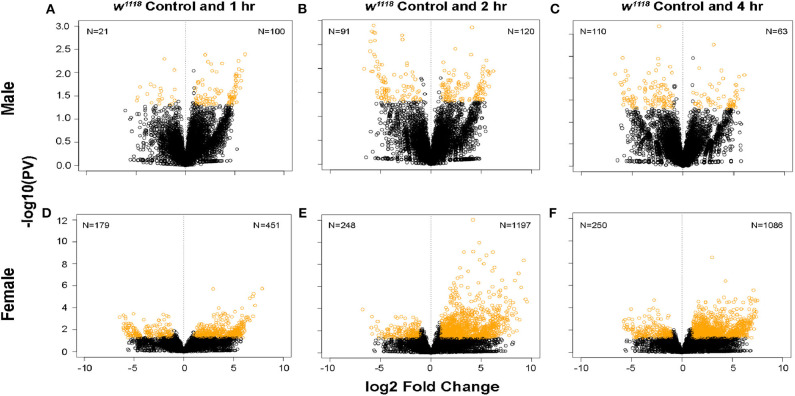
Gene expression changes after TBI in male and female flies. Volcano plots depicting log_2_ fold change and –log10(PV) of differentially expressed genes at 1, 2, and 4 hr after injury compared to control are indicated for males **(A–C)** and females **(D–F)**. The number of significantly upregulated and downregulated gene changes are indicated in yellow in each plot (|log_2_FC| > 1; *p* < 0.05). Females show more upregulated gene expression in response to injury than males.

Following brain injury, cellular cascades activate in response to the damage sustained by the primary and secondary effects of the insult (14). To identify genes involved in these pathways, we used 3′ mRNA-Seq to study genome wide gene expression changes between control and TBI flies in both sexes. The majority of TBI data identifying transcriptional changes has focused on investigating gene profiles several hours or days post-injury (29, 30, 33). In our study, we collected individual brains from control and 1-, 2-, and 4-hr post-injury (single strike) time points to capture gene expression changes within the immediate timeframe of TBI which may include primary and secondary effects. Differential gene expression analysis shows significant changes in both sexes after TBI (Figure 1). Gene expression changes in response to TBI were less pronounced in males (Figures 1A–C) as compared to females (Figures 1D–F). Females show more genes effected and more pronounced fold changes with the majority of the transcripts upregulated (|log FC| > 2; *p* < 0.05) across all time-points.

**Figure 2:**
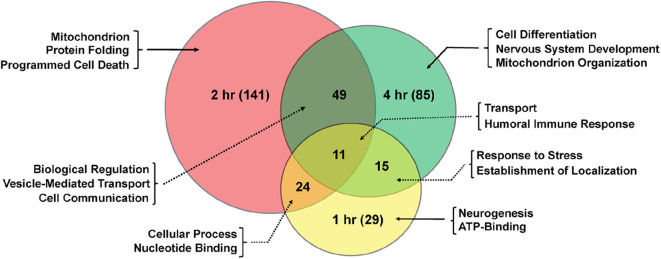
Shared gene ontology terms in females across time-points after injury. The Venn diagram shows significantly changed GO terms for females at 1, 2, and 4 hr after injury as well as overlap between time-points. The number of GO terms differentially regulated at each time-point is indicated in parenthesis.

Significant genes identified from mRNA-Seq were classified for their biological functions using DAVID (38, 45). Several gene ontology (GO) categories were changed in both sexes in response to TBI (Tables 1, 2 and Table S1). In females, the highest number of significant categories (FDR < 0.05) were altered 2 hr after injury (141 GO terms) (Figure 2). In addition to the 11 GO terms that overlapped between all 3 time-points, a large overlap was observed between processes at 2 and 4 hr after TBI (49 shared GO terms) (Figure 2). We observed significant changes in GO terms for “mitochondrion,” “neurogenesis,” “humoral immune response,” “programmed cell death,” “nervous system development” and “cell communication” in females (Figure 2, Table 1). Several studies on TBI have reported changes consistent with our findings including mitochondrial dysfunction (17, 46), immune activation (47, 48), apoptosis (49, 50), and activation of axonal regeneration after injury (51). Interestingly, genes involved in “nucleotide binding,” “neurogenesis,” “immune response,” and “mitochondrial translation” were among the processes that were significantly changed after TBI at all time-points. These GO categories are also among the top 10 terms altered after injury (Tables 1, 2). The largest impact on outcome after injury comes from damage to axons and accumulation of proteins involved in maintaining the cytoskeleton (22). Several studies have also focused on determining the link between TBI and later development of neurodegenerative disease like Alzheimer's (52), Parkinson's (53), and Amyotrophic lateral sclerosis (54). Dysfunction or accumulation of proteins like *tau* (22, 55), and amyloid precursor protein (*APP*) (56) have been implicated in TBI-mediated neurodegeneration. Surprisingly, we did not see any change in transcription of *tau* or *Appl* in our data (GSE140663: Differential gene expression and counts data). It is possible that alteration to protein or post-transcriptional regulation mediates the effects of these genes in response to TBI. However, we did see significant enrichment of “cytoskeleton,” “microtubule organization” and “axon transport” GO terms in females after injury, indicative of neurodegeneration after trauma. For males, although there are significant changes observed in gene expression related to “nervous system development,” “immune effector process,” and “neurogenesis,” there was no overlap seen between processes across any of the 3 time-points after injury (Table 2).

**Table 1:** Gene ontology terms significantly (FDR < 0.05) changed in females in response to traumatic brain injury.

**Rank**	**GOBPID**	**Term**	**Fold enrichment**	**FDR**
**(A) Selected GO terms differentially regulated in females after 1 hr of injury**				
1	GO:0022008	Neurogenesis	1.93	<0.01
2	GO:0044763	Single-organism cellular process	1.16	<0.01
3	UP_KEYWORDS	Nucleotide-binding	1.71	<0.01
4	GO:0000166	Nucleotide binding	1.48	<0.01
5	GO:1901265	Nucleoside phosphate binding	1.48	<0.01
6	UP_KEYWORDS	Atp-binding	1.73	<0.01
7	GO:0006790	Sulfur compound metabolic process	2.57	0.0110
8	GO:0051234	Establishment of localization	1.34	0.0112
9	GO:0009267	Cellular response to starvation	3.17	0.0120
10	GO:0006810	Transport	1.34	0.0133
11	GO:0032555	Purine ribonucleotide binding	1.51	0.0136
12	GO:0017076	Purine nucleotide binding	1.51	0.0137
13	GO:0097367	Carbohydrate derivative binding	1.45	0.0139
14	GO:0036094	Small molecule binding	1.40	0.0159
15	GO:0009987	Cellular process	1.08	0.0172
16	GO:0032553	Ribonucleotide binding	1.49	0.0179
17	GO:0032550	Purine ribonucleoside binding	1.49	0.0191
18	GO:0035639	Purine ribonucleoside triphosphate binding	1.49	0.0191
19	GO:0001883	Purine nucleoside binding	1.49	0.0209
20	GO:0032549	Ribonucleoside binding	1.49	0.0209
21	GO:0051186	Cofactor metabolic process	2.42	0.0214
22	GO:0001882	Nucleoside binding	1.48	0.0229
23	GO:0006950	Response to stress	1.41	0.0248
24	GO:0051188	Cofactor biosynthetic process	3.12	0.0264
25	GO:0051179	Localization	1.27	0.0272
26	GO:0044699	Single-organism process	1.09	0.0294
27	GO:0016887	Atpase activity	2.40	0.0370
28	GO:0044248	Cellular catabolic process	1.58	0.0441
29	GO:0006959	Humoral immune response	2.61	0.0462
**(B) Selected GO terms differentially regulated in females after 2 hr of injury**				
1	GO:0043207	Response to external biotic stimulus	2.30	<0.01
2	GO:0051707	Response to other organism	2.30	<0.01
3	UP_KEYWORDS	mRNA processing	2.28	<0.01
4	GO:0002440	Production of molecular mediator of immune response	2.83	<0.01
5	GO:0009617	Response to bacterium	5.31	<0.01
6	UP_KEYWORDS	Ribosomal protein	2.88	<0.01
7	GO:0045087	Innate immune response	3.38	<0.01
8	UP_KEYWORDS	Innate immunity	4.10	<0.01
9	UP_KEYWORDS	Immunity	3.95	<0.01
10	GO:0006952	Defense response	2.03	<0.01
19	GO:0019731	Antibacterial humoral response	4.34	<0.01
20	UP_KEYWORDS	Protein biosynthesis	3.25	<0.01
33	GO:0065007	Biological regulation	1.16	<0.01
56	UP_KEYWORDS	Oxidoreductase	1.48	<0.01
57	UP_KEYWORDS	Mitochondrion	1.76	<0.01
74	GO:0046907	Intracellular transport	1.48	<0.01
75	GO:0043038	Amino acid activation	2.97	<0.01
93	GO:0022008	Neurogenesis	1.42	0.0161
94	GO:0044700	Single organism signaling	1.20	0.0166
106	GO:0031349	Positive regulation of defense response	2.29	0.0211
107	GO:0007154	Cell communication	1.19	0.0216
122	GO:0023052	Signaling	1.18	0.0309
123	GO:0044765	Single-organism transport	1.23	0.0317
124	GO:0006906	Vesicle fusion	2.63	0.0324
130	GO:0008219	Cell death	1.47	0.0382
131	GO:0012501	Programmed cell death	1.49	0.0394
140	GO:0044262	Cellular carbohydrate metabolic process	1.62	0.0488
141	GO:0071496	Cellular response to external stimulus	1.92	0.0493
**(C) Selected GO terms differentially regulated in females after 4 hr of injury**				
1	UP_KEYWORDS	Ribonucleoprotein	2.69	<0.01
2	GO:0006950	Response to stress	1.55	<0.01
3	UP_KEYWORDS	Ribosomal protein	2.74	<0.01
4	GO:0006810	Transport	1.37	<0.01
5	GO:0051234	Establishment of localization	1.36	<0.01
6	GO:0044763	Single-organism cellular process	1.14	<0.01
7	GO:0051716	Cellular response to stimulus	1.29	<0.01
8	GO:0002181	Cytoplasmic translation	2.68	<0.01
9	GO:0042254	Ribosome biogenesis	2.15	<0.01
10	GO:0050896	Response to stimulus	1.22	<0.01
16	GO:0016192	Vesicle-mediated transport	1.58	<0.01
17	GO:0051179	Localization	1.24	<0.01
18	GO:0051649	Establishment of localization in cell	1.50	<0.01
29	GO:0009617	Response to bacterium	3.26	<0.01
30	GO:0007154	Cell communication	1.23	<0.01
31	GO:0044248	Cellular catabolic process	1.46	<0.01
37	GO:0033036	Macromolecule localization	1.38	<0.01
38	GO:0022008	Neurogenesis	1.48	<0.01
39	GO:0007005	Mitochondrion organization	1.70	<0.01
57	GO:0009991	Response to extracellular stimulus	1.82	0.0157
58	GO:0032543	Mitochondrial translation	2.34	0.0161
68	UP_KEYWORDS	Chaperone	2.73	0.0280
69	GO:0023052	Signaling	1.20	0.0284
73	GO:0000166	Nucleotide binding	1.24	0.0342
74	GO:1901265	Nucleoside phosphate binding	1.24	0.0342
81	GO:0019731	Antibacterial humoral response	2.86	0.0420
82	GO:0006959	Humoral immune response	1.99	0.0427
85	GO:0000902	Cell morphogenesis	2.63	0.0492

*GO terms were sorted based on FDR and ranked accordingly. Tables show selected GO terms changed in females after injury. GOBPID is the ID of the biological process in GO database*.

**Table 2:** Gene ontology terms significantly (FDR < 0.05) changed in males in response to traumatic brain injury.

**Rank**	**GOBPID**	**Term**	**Fold enrichment**	**FDR**
**(A) Selected GO terms differentially regulated in males after 1 hr of injury**				
1	GO:0002252	Immune effector process	6.56	0.0333
2	GO:0016485	Protein processing	9.00	0.0347
3	GO:0051604	Protein maturation	8.30	0.0464
**(B) Selected GO terms differentially regulated in males after 2 hr of injury**				
1	GO:0008104	Protein localization	2.47	<0.01
2	GO:0045184	Establishment of protein localization	2.72	<0.01
3	GO:0033036	Macromolecule localization	2.13	0.0118
4	GO:0015031	Protein transport	2.68	0.0146
5	GO:0009267	Cellular response to starvation	5.59	0.0215
6	GO:0007399	Nervous system development	1.63	0.0326
7	GO:0006950	Response to stress	1.78	0.0358
8	GO:0016070	RNA metabolic process	1.59	0.0386
9	GO:0051179	Localization	1.49	0.0448
10	GO:0050789	Regulation of biological process	1.33	0.0497
**(C) Selected GO terms differentially regulated in males after 4 hr of injury**				
1	UP_KEYWORDS	Transferase	2.25	<0.01
2	GO:1901605	Alpha-amino acid metabolic process	7.52	<0.01
3	GO:1901607	Alpha-amino acid biosynthetic process	12.40	<0.01
4	GO:0016740	Transferase activity	1.83	0.0115
5	GO:0051188	Cofactor biosynthetic process	6.50	0.0117
6	GO:0016053	Organic acid biosynthetic process	6.09	0.0164
7	GO:0044281	Small molecule metabolic process	2.13	0.0211
8	GO:0044711	Single-organism biosynthetic process	2.39	0.0436
9	GO:0044710	Single-organism metabolic process	1.63	0.0438
10	GO:0044283	Small molecule biosynthetic process	4.24	0.0454

*GO terms were sorted based on FDR and ranked accordingly. Tables show selected GO terms upregulated in males after injury. GOBPID is the ID of the biological process in GO database*.

The pattern of changes between male and female flies indicates that females show more transcriptional changes in response to TBI that continues up to 4 hr of injury. In males, however, the transcriptional response is more subtle at these early time-points.

### Immune Pathway Genes Are Differentially Regulated in Response to TBI

**Figure 3:**
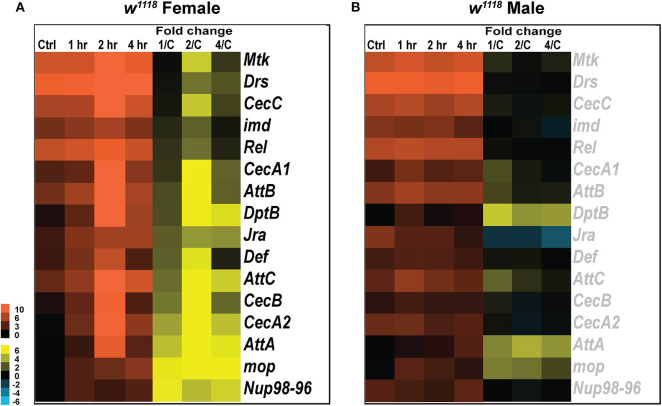
Immune gene expression changes in response to injury. Heatmaps depicting expression changes in immune function related genes for females **(A)** and males **(B)** at control, 1, 2, and 4 hr after injury. The orange scale represents average normalized counts for 3 replicates in each of the indicated groups. Yellow-blue scale shows fold change for each gene at 1, 2-, and 4-hr post-injury compared to control. 1/C, fold change at 1-hr compared to control; 2/C, fold change at 2-hr compared to control; and 4/C, fold change at 4-hr compared to control. All genes indicated in black font are significant (|log_2_FC| > 1, *p* < 0.05).

Multiple studies have explored the role of inflammatory processes and provided clues into the cell types and molecular pathways affected by TBI (30, 47, 48). TBI-related damage from secondary injuries due to activated immune response was shown previously in a similar fly model of head injury (57). In our analysis, we have also observed significant changes in transcript levels in several immune pathway genes in response to brain injury (Figure 3).

The *Drosophila* innate immune system is highly conserved with mammals and consists primarily of the Toll, Immunodeficiency (Imd) and Janus Kinase protein and the Signal Transducer and Activator of Transcription (JAK-STAT) pathways, which together combat fungal and bacterial infections (58, 59). Previous studies have explored activation of JAK-STAT (60) in response to injury but we did not see any change in gene expression associated with this pathway. We did, however, observe changes in transcript levels for genes involved in Toll, Imd pathway and JNK pathway as seen in other *Drosophila* TBI models (30, 57, 61). Although the genetic components for activation of Toll and Imd pathways are independent, induction of antimicrobial peptide genes like, *Drosomycin* (*Drs), Defensin (Def)*, and *Metchnikowin (Mtk)* depends on the activation of both pathways (62). In females, we observed a phasic response in several antibacterial and antifungal effector proteins like *Mtk, Drs, CecC, imd, Rel, Def*, and *CecB*, all upregulated 2 hr after injury but not significantly changed at 1 and 4 hr post-TBI (Figure 3A). *Mop*, a positive regulator of Toll-NF-κB signaling, is significantly upregulated at all timepoints in response to TBI. Several immune response genes including *AttA, AttC, CecA2*, and *Nup98-96* are induced in response to injury. Although significant at all time-points, a phasic change characterized by an increased upregulation of expression is observed in *AttA, AttC*, and *CecA2* at 2 h.

Unlike females, males have no significant transcript level changes in the antibacterial and antifungal genes assessed (Figure 3B). Although we see no significant induction of immune response after injury in males, there is consistently high levels of transcripts seen for some genes including *Rel* and *Drs*. *Rel*, a transcription factor involved in the immune deficiency pathway is highly expressed in both sexes at control and TBI conditions. Similarly, *Drs*, an antifungal peptide, is expressed at all conditions in both sexes but significantly induced only in females after injury. We also observed a phasic change in transcription of *repo*, a transcription factor specifically expressed in glial cells, in both sexes after injury (Data available in GSE140663: Differential gene expression and counts data). *Repo* transcription is significantly upregulated in females at 1 hr (log_2_FC: 3.10; *p* < 0.05) and 4 hr (log_2_FC: 4.02; *p* < 0.05) after injury with no significant change at 2 hr whereas in males, *repo* is significantly downregulated only at 2 hr (log_2_FC: −2.142; *p* < 0.05) after injury. *Drs, DptB, Mtk*, and *AttA* expression was tested by qRT-PCR for both males and females (Table S2).

These data suggest that immune response varies in males and females post-TBI. Interestingly, females exhibit a phasic change in immune pathways with induction of some genes 2 hr after trauma but no significant change at 1 and 4 hr. Phasic activation of immune response genes has previously been observed in transcriptional studies from a mixture of male and female flies inflicted with TBI within 1–8 hr after injury (29). Thus, similar studies over longer time-points would be helpful to deduce if this pattern is repeated beyond the time-points assessed.

### TBI Affects Transcript Levels of Genes Related to Mitochondrial Transport and Oxidative Phosphorylation

**Figure 4:**
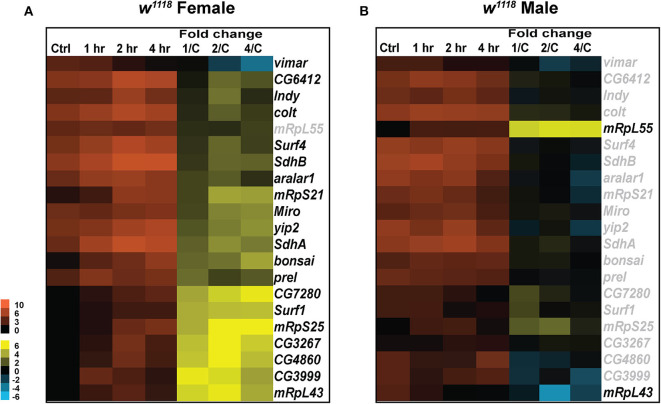
Mitochondrial gene expression changes after injury. Heatmaps depicting expression changes in mitochondria related genes for females **(A)** and males **(B)** at control, 1, 2, and 4 hr after injury. Orange scale represents the average of normalized counts for 3 replicates in each group indicated above. Yellow-blue scale shows fold change for each gene at 1, 2-, and 4-hr post-injury compared to control. 1/C, fold change at 1-hr compared to control; 2/C, fold change at 2-hr compared to control; and 4/C, fold change at 4-hr compared to control. All genes indicated in black font are significant (|log_2_FC| > 1, *p* < 0.05).

Mitochondria are subcellular organelles required for several metabolic processes and energy generation by oxidative phosphorylation (63). Mitochondria are present in all cell types and organ systems but differ in morphology and quantity suggesting tissue and system-specific roles (64). Injury to mitochondria leads to oxidative stress, subsequent apoptosis and decreased cellular energy (17). These changes impair neurologic functions, as commonly observed not only in TBI but also in other neurodegenerative diseases (46). We, therefore, looked at changes in expression of genes related to mitochondria and oxidative phosphorylation in *Drosophila* (Figure 4).

In female flies, a significant increase in transcripts was observed for *Miro* (vital for mitochondrial homeostasis and microtubule-based mitochondrial transport) and *prel* (which contributes to the integrity of mitochondrial structures and the activity of respiratory chain complex IV) (Figure 4A). Transcripts for *vimar*, a guanine nucleotide exchange factor for *Miro*, were significantly decreased post-TBI. *Vimar* has been previously shown to increase mitochondrial fission in *Drosophila* (65) so change in expression of *vimar* and *miro* post-TBI could be indicative of alteration in mitochondrial dynamics in response to injury. We also observed a significant increase in transcripts in the SLC25 family of mitochondrial transporters including *colt* and *aralar1*. SLC25 family of transport proteins shuttle metabolites, nucleotides and cofactors across the inner mitochondrial membrane and are essential for energy conversion and cell homeostasis (66). An increase in response to injury in these genes might be mediated through the increase in cellular energy demands for repair of damage sustained by TBI. Additionally, we have observed a significant increase in transcripts for genes involved in the *Drosophila* oxidative phosphorylation system including *Surf1* (involved in the assembly of the mitochondrial respiratory chain Cytochrome Oxidase), *SdhA* and *SdhB* (encoding the iron-sulfur cluster-containing subunit of the succinate dehydrogenase complex, which oxidizes succinate to fumarate) after injury. The oxidative phosphorylation system drives the synthesis of ATP; therefore, it is not surprising to find an increase in gene expression related to this system post-injury to upregulate cell capacity and generate more ATP. In addition, upregulation of genes involved in mitochondrial biogenesis (*mRpS21, mRpS25* and *mRpL43*) also occurs post-TBI. Mitochondrial biogenesis can be altered as part of a concerted cellular response to metabolic changes that demand more ATP output and increase in functional mitochondria (67). This is important to TBI pathology as mitochondrial dysfunction is associated with increased reactive oxygen species (ROS) production, an effect of mitochondrial dysfunction and contributes to toxicity (68).

Similar to the differences seen in immune response between sexes, males have few significant changes for mitochondria related genes (Figure 4B). Only two mitochondrial biogenesis genes *mRpL55*, increased transcripts at all time points, and *mRpL43*, decreased transcripts at 2 hr, show differential expression in males. In females *mRpL55* is unchanged while *mRpL43* is upregulated at all time points. *mRpL55* expression was confirmed by qRT-PCR for both sexes (Table S2). This data demonstrates that TBI influences expression of genes involved in mitochondrial activity and oxidative phosphorylation significantly in females at early TBI timepoints with few changes seen in males. There is evidence of increased mitochondrial biogenesis 24 hr after TBI in male mice (69), so the disparity in transcription of *mRpL55* and *mRpL43* in male flies is surprising. For both sexes, it is possible that transcription of other biogenesis related genes may increase at later time-points.

### Mitochondrial Stress Is Increased in Response to TBI

Mitochondria play an important role in maintaining cellular energy homeostasis through oxidative phosphorylation system (70). Highly reactive oxygen species are byproducts typically generated during such respiration and metabolism processes (70). In healthy conditions, endogenous antioxidants like superoxide dismutase and glutathione molecules inhibit production of ROS (71). Under physiological stress conditions such as brain injury, ROS production increases dramatically causing significant cell damage (72). Impaired mitochondrial function as a result of excessive ROS is also seen in neurodegenerative diseases like Alzheimer's, Parkinson's, Huntington's, and tauopathies (73, 74). Mitochondrial dysfunction coupled with increased ROS and decreased ATP production is commonly seen in secondary damage to TBI (17). Thus, monitoring mitochondrial turnover is important considering its essential role in health and disease.

**Figure 5:**
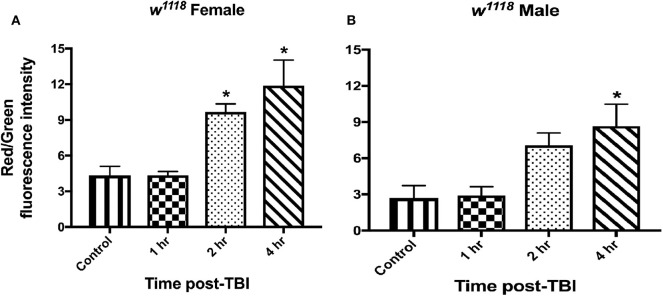
Mitochondrial stress increases in both sexes after injury. Measurement of mitochondrial oxidation in female **(A)** and male **(B)** flies at control and 1, 2, and 4-hr post-injury using the *UAS-MitoTimer* reporter shows increased oxidation after injury. In both sexes, mitochondrial oxidation is significantly (*p < 0.05; One-way ANOVA with Dunnett test) increased post-TBI. Overall, males show lower levels of red/green fluorescence intensity ratio than females at each time-point. For each condition, 3 replicates of 10 brains each were assessed and mean ± SEM were plotted.

To assess mitochondrial health *in vivo*, we made use of the *MitoTimer* reporter gene (75, 76). *MitoTimer* encodes a DsRed mutant (DsRed-E5) that fluoresces green when mitochondria are newly synthesized and shifts irreversibly to red upon oxidation (75). The maturation from green to red is unaffected by pH, ionic strength or protein concentration, but is affected by light, temperature and oxygen exposure (77). In this study, we expressed *UAS-MitoTimer* using the pan-neural driver *elav-Gal4* and measured fluorescence at control and TBI conditions. The ratio of red/green fluorescence intensity is a measure of mitochondrial stress (76). For both sexes, we observed no change in mitochondrial oxidation after 1 hr of injury (Figure 5, Figure S1). We saw a significant increase in red/green ratio after 2 and 4 hr of injury in females (Figure 5A, Figure S1A) whereas males show significant change only after 4 hr (Figure 5B, Figure S1B). Although this indicates increased mitochondrial turnover and oxidation in both sexes, it also suggests a delayed response in males. In a previous study, we have also shown significant increase in COX activity and decrease in ATP production 24 hr after TBI (33).

### TBI Impairs Locomotor Activity and Climbing Ability in *Drosophila*

**Figure 6:**
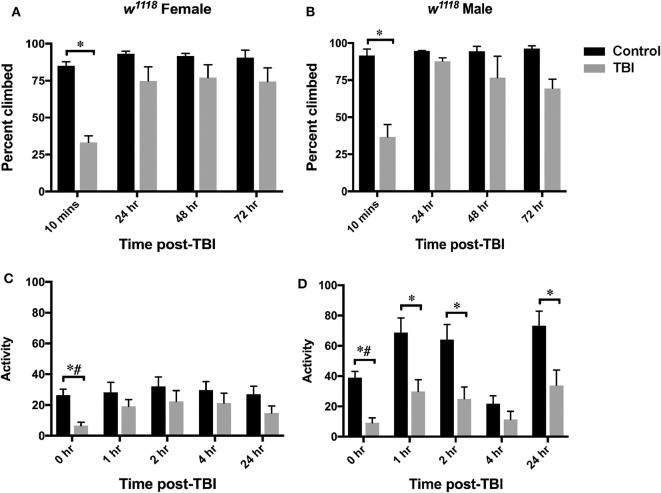
Motor function is affected after injury in both sexes. Plots show defects in climbing ability (top-panel) and locomotor activity (bottom-panel) in females **(A,C)** and males **(B,D)** after TBI. Climbing ability is indicated as percent climbed for an average of 3 replicates (20 flies per replicate) and assessed at control, 10 min, 24-, 48-, and 72- h after injury. Significant decrease in percent climbed is observed for both sexes at 10 min after injury (*p* < 0.05 from mixed design ANOVA with Tukey). Locomotor activity at control, 1-, 2-, 4-, and 24-hr post-injury is shown as an average of activity for at least 20 flies in each group. A significant decrease in locomotor activity is observed for both sexes after TBI (*p < 0.05 from LSD and ^#^*p* < 0.05 from Bonferroni).

Behavioral effects like loss of motor skills, coordination, and balance impairment are commonly observed post-TBI in experimental models and also in clinical patients (25). Mild TBI in mice is shown to alter diurnal locomotor activity and response to light (78). A comparison of mobility in people who suffered a moderate to severe TBI compared to controls suggests that even if TBI patients seem to have generally recovered their locomotor abilities, deficits can persist (79). Flies also exhibit ataxia and incapacitation not attributable to damaged legs and wings after injury (27). To assess the extent of injury in movement behavior after TBI, we analyzed climbing ability and locomotor activity at control and TBI conditions for both male and female flies (Figure 6). The climbing assay employs tapping of the vials to cluster flies at the bottom, thus subjecting them to a mechanical stimulus which has a rapid kinetic effect on flies. Locomotion behavior, however, assesses motor coordination in the absence of a stimulus and climbing-independent movement.

Normally, when stimulated by tapping to the bottom of the vial, flies rapidly climb to the top and stay there. This behavior in *Drosophila* is called negative geotactic response and has been studied in fly models of neurodegeneration to identify molecules involved in fine motor control (80). We used a climbing assay to assess defects in this response after injury by tapping them to the bottom of vial and recording the number of flies that cross 70% height of the vial in 15 sec. We observed a significant decrease (mixed design ANOVA with Tukey) in climbing ability only at 10 min after injury for both females (Figure 6A) and males (Figure 6B) implying similar recovery in climbing ability for both sexes (Figures 6A,B).

Locomotor activity was assessed in flies using the Drosophila activity monitoring system (TriKinetics Inc., Waltham, MA). Adult flies were placed in monitors immediately after being inflicted with brain injury and activity data was collected for 48 hr. We observed a significant decrease in activity in both sexes immediately after TBI at 0 hr (repeated measures ANOVA with LSD and Bonferroni) (Figure 6, Figure S2). Females show some recovery starting at 1 hr and continuing through 24 hr (Figure 6C, Figure S2A), but males have significantly decreased activity up to 24 hr (only with LSD comparison) (Figure 6D, Figure S2B). Overall, females have lower locomotor activity than males at all time-points including control flies. We saw no change after 24 hr (data not shown).

These data suggest that although both sexes exhibit motor defects in response to TBI, females show faster recovery than males.

## Discussion

Sex as a confounding biological variable to TBI outcome has not been widely considered in previous transcriptomic studies (81–85). In flies, genome-wide mRNA expression profiles were studied in whole male flies to compare age and diet related mechanisms that contribute to injuries after TBI (27, 29) and transcriptional changes at 1-, 3-, and 7-days post-injury in whole heads using Translating Ribosome Affinity Purification and Sequencing (TRAP-seq) (30). We have also previously shown selective intron retention in genes associated with tricarboxylic acid (TCA) cycle 24 hr post-TBI in whole heads from male and female flies pooled together after 1- or 2-strikes (27, 33). Since the *Drosophila* brain occupies only a very small part of the head, about 14% dry weight (86), the previous whole head studies may include transcript information derived from non-brain tissue (30, 33). In this present study, 3′ mRNA libraries were constructed from isolated adult male and female brains before and after TBI to examine sex dependent outcomes post-injury in the fly brain (27). Our results suggest that, overall, *Drosophila* females exhibit stronger gene expression changes in response to TBI than males. Although we see sex differences in gene transcription, the cause remains unclear. The presence of metabolic tissues or sex-specific gene expression could be potential factors for these differences and require further study. We also assessed motor function and mitochondrial health in both sexes after injury and observed that although both sexes show motor defects and increased mitochondrial oxidation, males exhibit subtle changes in both the number of altered transcripts and magnitude of differential gene expression post-injury than females.

### *Drosophila* Males Show Weaker Immune Response After TBI

Immune response to injury in the brain is a key mediator in recovery, and progressive impairments become apparent when compromised (20, 87). Many groups have reported activation of neuroinflammatory response after TBI, which is also recognized as a key player in recovery (88, 89) but very few studies have reported sex divergence of TBI-mediated neuroinflammation. In humans, gender-differences in immunity are well-established (90, 91) as seen from the fact that females produce more severe immune reactions and have a higher incidence of autoimmune diseases, which may result from the influence of sex hormones on the immune system (92). In *Drosophila* too, dichotomy between the sexes in the gene mRNA levels of the antifungal genes *Drs* and *Mtk* has previously been observed (90). In our study, we saw upregulation of several genes involved in Toll and Imd pathways in females with no significant changes observed in males (Figure 3). Several of the genes implicated in the Toll pathway have increased transcripts in females but no change in males. Transcripts of other immune response genes (*Def, Attacins, Cecropins*) that are seen to be increased in females in our model, were also seen to be higher in males from a *Drosophila* closed head injury model 1 and 3- days post-injury (30). Since we observed no transcriptional changes in males up to 4 hr after injury, one possible explanation which would require future study is that the immune response to injury in *Drosophila* males is not as immediate as in females. It is also important to consider that some of the immune effects could result from the sex-dependent response to full body trauma.

### Bimodal Activation of Immune Response After TBI

The neuroinflammatory reaction that follows TBI is a result of the interactions between several immune pathways (48). Here, we observed significantly increased transcripts only at 2 hr for *Mtk, Drs, CecC, imd*, and *Rel* in females (Figure 3). It is not known whether the expression profile of these genes continues to remain unchanged after 4 hr or another elevated response would be observed in additional timepoints. Considering that there are several immune genes changed only in females after injury, it is possible that the immune response in females is induced immediately after trauma and plateaus within a few hours. In males, however, it is likely that the response initiates after a few hours and continues to remain upregulated for a few days post-trauma, as seen in a male mouse model of controlled cortical impact where microglia decreased 1 day after injury and increased at 1 and 2 weeks post-injury (93). In general, immune response is intended to promote neuroprotection and recovery, but when dysregulation arises, it can become maladaptive (94). Therefore, it is important to better understand the timing and dynamic changes in immune response after TBI. Altogether, our results show that the immune response in female and male *Drosophila* are not identical and result from complex interplay of many factors including a phasic change in gene expression.

### Sex-Differences in Mitochondrial Gene Expression in Response to TBI

It is becoming increasingly apparent that mitochondrial metabolism is also sexually dimorphic (95). Sex specificities in mitochondrial biogenesis, ATP production, oxidative phosphorylation activities, oxygen consumption, ROS production and calcium uptake have been observed in different tissues from rodents and humans (96–99). In our analysis, we observed higher transcription of genes involved in oxidative phosphorylation, mitochondrial protein transporters, and mitochondrial translation in females than males after injury (Figure 4). At baseline, the expression of several genes is similar in both sexes, but transcription levels exhibit diverse changes in response to injury. These findings match reports in a pediatric TBI model of rats, where mitochondrial activity is higher in females after injury (100). However, we did observe significant upregulation in mitochondrial turnover in both sexes (Figure 5, Figure S1), indicating gender similarities in clearance of damaged mitochondria. In the incidence of stroke and neurodegenerative diseases, sex differences in mitochondrial protective effects of estrogen were also identified (101). Although, it has not been extensively studied in *Drosophila*, the possibility of sex-specific genes regulating hormones which in turn influence the response to brain injury cannot be ruled out. Emerging evidence also suggests that mitochondria provide a platform for signaling pathways involved in immune response mainly through transcriptional regulation of inflammatory chemokines/cytokines and their maturation of inflammasomes (102, 103). It is, therefore, not surprising that females show increased transcript levels of mitochondrial and immune genes in response to TBI in our study. It is, however, yet to be established, what sex-specific genes or hormones contribute to a possible delayed response in males but an immediate response in females.

### Behavioral Defects Differ Between Males and Females After Injury

Individuals with traumatic brain injury often experience lasting locomotor deficits and impaired motor coordination (79). Sexual dimorphism in locomotor activity of *D. melanogaster* has been widely studied (104–106). We also observed activity differences in our study where male flies exhibit reduced locomotion, but females do not show similar changes post-injury (Figure 6, Figure S2). This disparity in recovery after TBI suggests that impairments cannot be fully attributed to physical damages but may also involve sex-specific gene changes. Interestingly, we observed significant increase in *Tip60* transcripts after injury in females. In a *Drosophila* model of Alzheimer's, increasing levels of *Tip60* histone acetyltransferase rescued axonal transport and larval motor function defects (107). Therefore, it is possible that certain locomotor or movement associated gene changes in females are protective and contribute toward faster recovery than males.

## Conclusion

In this study, we looked at changes in gene expression and motor function in response to TBI in *w*^1118^ male and female flies fed standard diet. In summary, we found an effect of biological sex on brain injury response and outcome. Throughout post-TBI assessment, we saw elevated immune genes with peak transcript levels occurring at 2 hr post-TBI in females. Our findings offer insights into the complexities of the different outcomes of brain injury that can be further explored in the development of treatment and management strategies to improve outcomes.

## Data Availability Statement

Gene expression data are available in the GEO database under accession number GSE140663. Table S1 contains gene ontology analyses for significant gene transcription changes.

## Author Contributions

DR and KG are senior authors and conceived the project. ES and KG conceptualized the content. ES performed the data analysis and wrote the manuscript. KG edited the manuscript and assisted with data analysis.

## Conflict of Interest

The authors declare that the research was conducted in the absence of any commercial or financial relationships that could be construed as a potential conflict of interest.

## Supplementary Material

Figure S1 Individual data points for MitoTimer assay. Plots show individual data points for MitoTimer reporter assay for females **(A)** and males **(B)** at control and 1, 2, and 4 hr post-TBI. Three replicates of 10 brains each were assessed for every time-point and mean ± SEM were plotted.

Figure S2 Individual data points for locomotor activity. Plots show individual data points for locomotor activity for females **(A)** and males **(B)** at control and 1, 2, 4, and 24 hr post-TBI (*n* > 20). Each data point represents average activity of the fly in the 30-min bin before the specified time-point.

Table S1 Gene Ontology tables for males and females.

Table S2 qRT-PCR data for select genes.
