# Supplementary figures and images for: Drosophila Exhibit Divergent Sex-Based Responses in Transcription and Motor Function After Traumatic Brain Injury
Source: Front Neurol. 2020 Jun 19;11:511. doi: 10.3389/fneur.2020.00511 (PMC7316956; doi:10.3389/fneur.2020.00511)

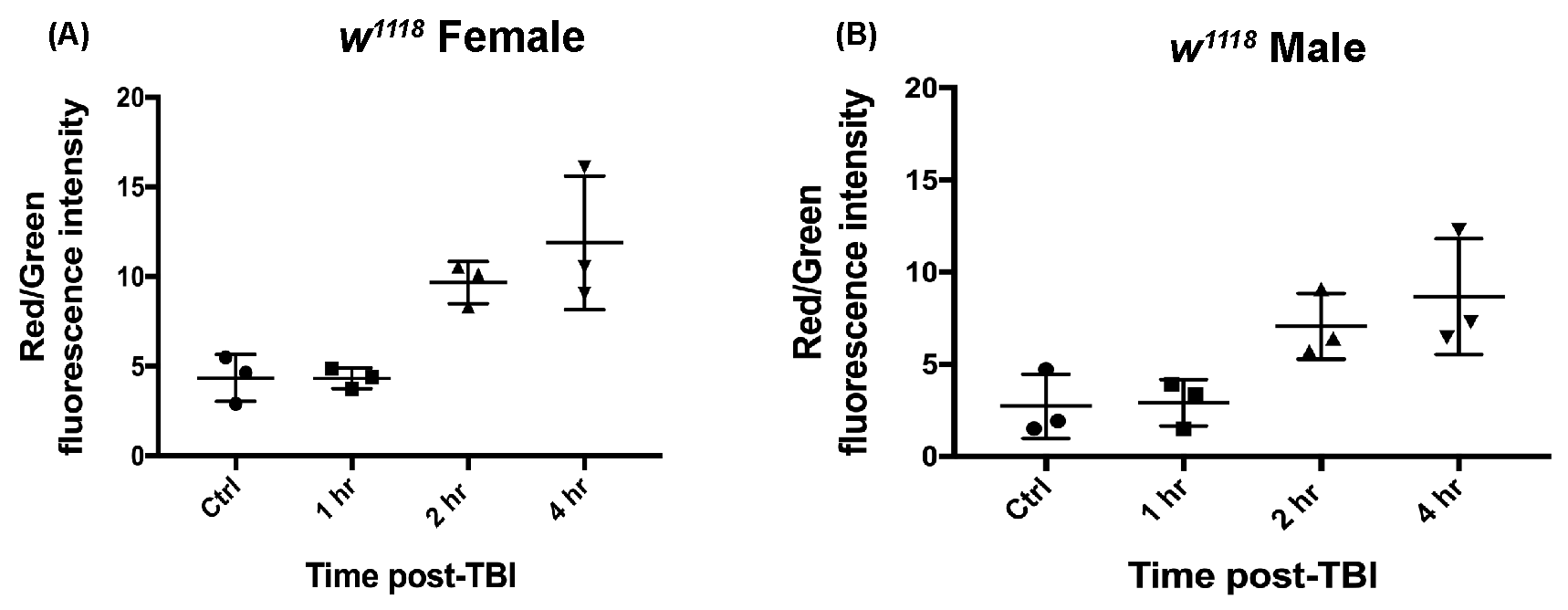

Supplement: Figure S1 — Individual data points for MitoTimer assay. Plots show individual data points for MitoTimer reporter assay for females (A) and males (B) at control and 1, 2, and 4 hr post-TBI. Three replicates of 10 brains each were assessed for every time-point and mean ± SEM were plotted. [file Image_1.TIF]

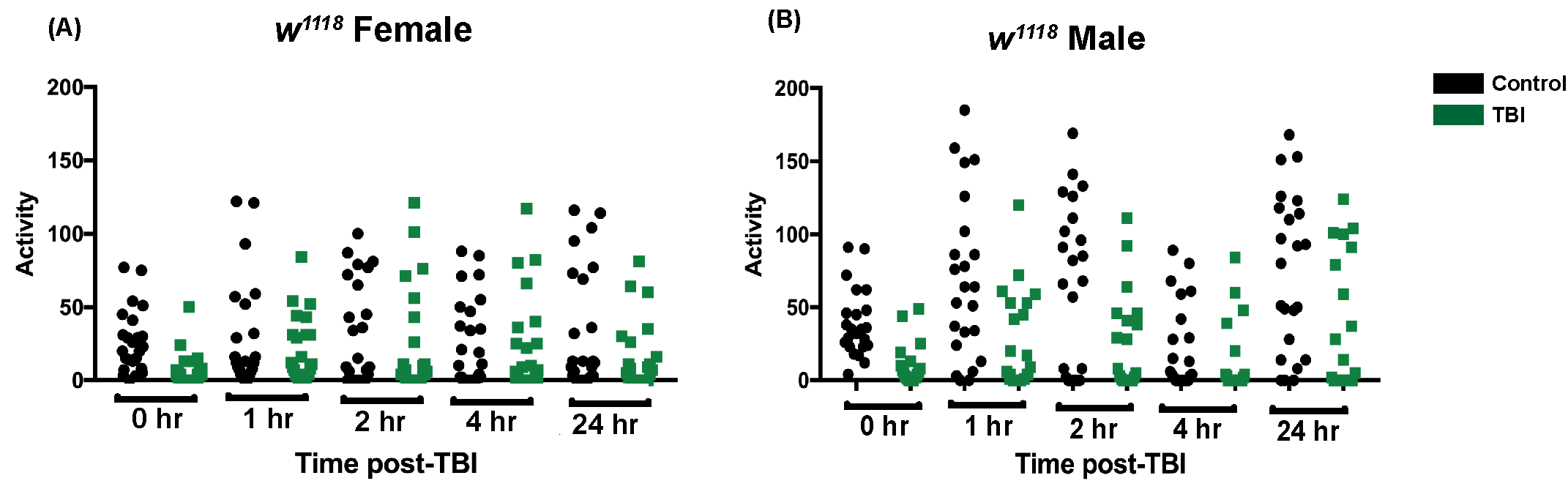

Supplement: Figure S2 — Individual data points for locomotor activity. Plots show individual data points for locomotor activity for females (A) and males (B) at control and 1, 2, 4, and 24 hr post-TBI (n > 20). Each data point represents average activity of the fly in the 30-min bin before the specified time-point. [file Image_2.TIF]
